# Strengthening guideline contextualization in the WHO European Region

**DOI:** 10.2471/BLT.24.291779

**Published:** 2024-08-23

**Authors:** Marge Reinap, Naomi Limaro Nathan, Natasha Azzopardi Muscat, Kaja-Triin Laisaar, Urmeli Katus, Holger J Schünemann

**Affiliations:** a World Health Organization Regional Office for Europe, Marmorvej 51, 2100 Copenhagen, Denmark.; b Institute of Family Medicine and Public Health, University of Tartu, Tartu, Estonia.; c Clinical Epidemiology and Research Center, Humanitas Research Hospital, Milan, Italy.

## Abstract

The World Health Organization (WHO) plays an important role in developing evidence-based and ethically sound guidelines to assist health workers, programme managers and policy-makers, particularly in countries with limited capacities to create their own. While the development of these guidelines follows rigorous methods, contextualizing recommendations is often necessary to ensure their applicability, feasibility and acceptability at the country level. The adaptation and adoption of global guidelines should happen in a transparent, systematic and participatory manner to maintain credibility while ensuring the ownership necessary for implementation. Here, we present an example from Estonia that showcases the process, requirements and outcomes of implementing WHO guidelines through effective contextualization. The work in Estonia showed that contextualization can shorten guideline development time and reduce costs. To support countries in contextualizing guidelines, including those developed by WHO, to local contexts while maintaining trustworthiness and relevance, the WHO Regional Office for Europe has developed a handbook based on the GRADE-Adolopment approach to guide this process. Furthermore, a rapid assessment of 21 of the 53 Member States in the WHO European Region revealed that many countries need guidance and support to build capacity for contextualizing guidelines. To address the capacity gaps, we suggest a way forward that encompasses four areas of further work: standardizing methods; institutionalizing guideline programmes and initiatives; promoting continuous and shared learning; and providing support and identifying resources. Strengthening countries’ capacities to contextualize global guidelines is crucial and will become especially relevant during future health threats, such as pandemics, climate change and conflict situations.

## Résumé

L'Organisation mondiale de la Santé (OMS) joue un rôle crucial dans l'élaboration de lignes directrices éthiques et fondées sur des données factuelles qui viennent soutenir le personnel de santé, les gestionnaires de programmes et les responsables politiques, en particulier dans les pays qui ne disposent pas de capacités suffisantes pour développer les leurs. Bien que la mise au point de ces lignes directrices suive des méthodes rigoureuses, il est souvent indispensable de contextualiser les recommandations pour les rendre applicables, faisables et acceptables à l'échelle nationale. L'adaptation et l'adoption de lignes directrices mondiales doivent s'effectuer en toute transparence, de manière systématique et participative, en vue de préserver leur crédibilité tout en assurant le niveau d'implication nécessaire à leur déploiement. Ce document s'appuie sur un exemple provenant d'Estonie pour illustrer le processus, les exigences et les résultats liés à la mise en pratique de lignes directrices émises par l'OMS grâce à une contextualisation efficace. Le travail réalisé en Estonie a montré que cette contextualisation pouvait réduire le temps consacré au développement de la ligne directrice et faire baisser les coûts. Afin d'aider les pays à adapter les lignes directrices, notamment celles élaborées par l'OMS, à chaque contexte local tout en conservant leur fiabilité et leur pertinence, le Bureau régional de l'OMS pour l'Europe a rédigé un manuel qui s'inspire de l'approche GRADE-Adolopment pour guider ce processus. En outre, une évaluation rapide de 21 des 53 États Membres de la Région européenne de l'OMS a révélé que nombre d'entre eux avaient besoin d'assistance et de conseils pour développer leurs capacités de contextualisation des lignes directrices. Nous suggérons, pour combler les lacunes, une stratégie constituée de quatre axes pour la poursuite des travaux: la standardisation des méthodes, l'institutionnalisation des initiatives et programmes relatifs aux lignes directrices, la promotion d'un apprentissage commun et continu, ainsi que la mise à disposition d'un soutien et l'identification des ressources. Renforcer les capacités des pays à contextualiser les lignes directrices mondiales est essentiel et revêtira une importance particulière face à de futures menaces sanitaires telles que les pandémies, le changement climatique et les situations de conflit.

## Resumen

La Organización Mundial de la Salud (OMS) desempeña una función importante en la elaboración de directrices basadas en evidencias y sólidas desde el punto de vista ético para ayudar al personal sanitario, los gestores de programas y los responsables de formular políticas, sobre todo en países que tienen una capacidad limitada para crear sus propias directrices. Aunque la elaboración de estas directrices sigue métodos rigurosos, suele ser necesario contextualizar las recomendaciones para asegurar su aplicabilidad, viabilidad y aceptabilidad a nivel nacional. La adaptación y la adopción de directrices mundiales deben realizarse de forma transparente, sistemática y participativa para mantener la credibilidad y asegurar al mismo tiempo la apropiación necesaria para su implementación. En este documento, se presenta un ejemplo de Estonia que muestra el proceso, los requisitos y los resultados de la aplicación de las directrices de la OMS mediante una contextualización efectiva. El trabajo realizado en Estonia demostró que la contextualización puede acortar el tiempo de elaboración de las directrices y reducir los costes. Para ayudar a los países a adaptar las directrices, incluidas las elaboradas por la OMS, a los contextos locales, manteniendo al mismo tiempo su fiabilidad y pertinencia, la Oficina Regional para Europa de la OMS ha elaborado un manual basado en el enfoque GRADE-Adolopment para orientar este proceso. Además, una evaluación rápida de 21 de los 53 Estados Miembros de la Región de Europa de la OMS reveló que muchos países necesitan orientación y apoyo para crear capacidad a fin de contextualizar las directrices. Para abordar las carencias de capacidad, se sugiere una vía de avance que abarca cuatro áreas de trabajo ulterior: estandarizar métodos, institucionalizar programas e iniciativas de directrices, promover el aprendizaje continuo y compartido, y proporcionar apoyo e identificar recursos. El refuerzo de las capacidades de los países para contextualizar las directrices mundiales es crucial y será de especial relevancia durante futuras amenazas para la salud, como las pandemias, el cambio climático y las situaciones de conflicto.

## ملخص

تلعب منظمة الصحة العالمية (WHO) دورًا مهمًا في تطوير المبادئ التوجيهية القائمة على الأدلة والسليمة أخلاقيًا، وذلك لمساعدة العاملين في المجال الصحي، ومديري البرامج، وواضعي السياسات، وخاصة في الدول ذات القدرات المحدودة على إنشاء مبادئها الخاصة بها. وبينما يكون تطوير هذه المبادئ التوجيهية مترتبًا على أساليب صارمة، فإن صياغة التوصيات غالبًا ما تكون ضرورية لضمان قابلية تطبيقها، وجدواها، وقبولها على مستوى الدولة. إن تكييف المبادئ التوجيهية العالمية وتبنيها يجب أن يحدث بطريقة شفافة ومنهجية وبطريقة مشتركة للحفاظ على المصداقية مع ضمان الملكية الضرورية للتنفيذ. ونحن هنا نقدم مثالاً من إستونيا يوضح كل من العملية، والمتطلبات، ونتائج تنفيذ المبادئ التوجيهية لمنظمة الصحة العالمية من خلال الصياغة الفعالة. وقد أظهر العمل في إستونيا أن الصياغة يمكنها أن تقصّر وقت تطوير المبادئ التوجيهية، وتقلل التكاليف. لدعم الدول في صياغة المبادئ التوجيهية لتكون مناسبة لسياقات محلية مع الحفاظ على الثقة والأهمية، بما في ذلك المبادئ التي طورتها منظمة الصحة العالمية، فقد وضع المكتب الإقليمي لمنظمة الصحة العالمية لدى أوروبا كتيبًا يستند إلى أسلوب GRADE-Adolopment لتوجيه هذه العملية. وفضلاً عن ذلك، فإن التقييم السريع لـ 21 دولة من إجمالي 53 دولة عضو في المكتب الإقليمي لمنظمة الصحة العالمية لدى أوروبا، قد كشف أن العديد من الدول تحتاج إلى التوجيه والدعم لبناء القدرة على صياغة المبادئ التوجيهية. ولمعالجة فجوات القدرات، فإننا نقترح طريقة للمضي قدمًا تشمل أربعة مجالات من العمل الإضافي، وهي: التوحيد القياسي للأساليب، وإضفاء الطابع المؤسسي على برامج ومبادرات المبادئ التوجيهية، والارتقاء بالتعلم المستمر والمشترك، وتقديم الدعم وتحديد الموارد. إن تعزيز قدرات الدول على صياغة المبادئ التوجيهية يُعد أمرًا حيويًا، وسيصبح صائبًا على نحو خاص خلال التهديدات الصحية المستقبلية، مثل الجوائح، وتغير المناخ، ومواقف النزاع.

## 摘要

世界卫生组织（世卫组织）在制定合乎道德的循证指导方针以协助卫生工作者、方案管理人员和政策制定者开展工作方面发挥着重要作用，特别是对难以独立制定指导方针的国家来说。虽然在制定这些指导方针时采用了严格的方法，但为了确保其在国家层面的适用性、可行性和可接受性，通常会建议对其进行情境化处理。应以透明、系统和参与性的方式调整和采用全球性指导方针以维持可信度，同时确保实施时体现必要的自主权。我们在此以爱沙尼亚为例，展示通过有效的情境化处理实施世卫组织指导方针的过程、要求和成果。爱沙尼亚的相关工作表明，情境化处理可以缩短制定指导方针所需的时间并降低成本。为了支持各国在维持可信度和相关性的同时根据当地实际情况对指导方针（包括世卫组织制定的指导方针）进行情境化处理，世卫组织欧洲区域办事处基于 GRADE-Adolopment 方法编写了一份手册，以指导这一流程。此外，针对世卫组织欧洲区域 53 个成员国中的 21 个国家开展的快速评估显示，许多国家需要在指导和支持下方可获得对指导方针进行情境化处理的能力。为了解决能力差距，我们提出了今后工作的努力方向，即进一步强化四个领域：方法标准化、指导方针相关计划和倡议制度化、推广持续和共享学习，以及提供支持和确定资源。加强各国对全球性指导方针进行情境化处理的能力至关重要，在未来出现流行病、气候变化和冲突局势等健康威胁时其作用尤为明显。

## Резюме

Всемирная организация здравоохранения (ВОЗ) играет важную роль в разработке научно обоснованных и этически оправданных руководств в помощь медицинским работникам, руководителям программ и политикам, особенно в странах с ограниченными возможностями по созданию собственных руководств. Несмотря на то что при разработке этих руководств используются строгие методы, для обеспечения их применимости, осуществимости и приемлемости на уровне стран часто требуется контекстуализация рекомендаций. Адаптация и принятие глобальных руководств должны осуществляться на прозрачной, систематической и коллективной основе для поддержания доверия и обеспечения необходимой для реализации ответственности. В этой статье приводится пример Эстонии, который демонстрирует процесс, требования и результаты внедрения рекомендаций ВОЗ с помощью эффективной контекстуализации. Результаты работы в Эстонии свидетельствуют о том, что контекстуализация может сократить время разработки рекомендаций и снизить затраты. Чтобы помочь странам в адаптации руководств, в том числе разработанных ВОЗ, к местным условиям при сохранении надежности и актуальности, Европейским региональным бюро ВОЗ разработано учебное пособие, основанное на методике GRADE-Adolopment, для руководства этим процессом. Кроме того, по результатам экспресс-оценки 21 из 53 государств-членов Европейского региона ВОЗ выяснилось, что многие страны нуждаются в направлении и поддержке усилий для создания возможностей по контекстуализации руководств. Для устранения пробелов в области возможностей предложен путь, включающий четыре направления дальнейшей работы: стандартизацию методов, институционализацию программ и инициатив по разработке рекомендаций, содействие непрерывному и совместному обучению, а также оказание поддержки и определение ресурсов. Укрепление потенциала стран по контекстуализации глобальных рекомендаций имеет решающее значение и станет особенно актуальным в случаях прогнозируемых в будущем угроз здоровью, таких как пандемии, изменение климата и конфликтные ситуации.

## Introduction

The World Health Organization (WHO) has the mandate to develop guidelines and provide recommendations under its normative and global standard-setting function.^1^ These guidelines assist health workers, programme managers and policy-makers in making appropriate health-care decisions, and are especially important for countries with limited capacities to develop their own guidelines. While the guidelines development follows gold-standard methods, including the Grading of Recommendations Assessment, Development and Evaluation (GRADE) approach,^2^ additional processes such as contextualization (Box 1) are often required to ensure their implementation is applicable, equitable, feasible and acceptable for use at the country level.

Box 1 **Definition of contextualization of guidelines^3^^,^^4^**

The process of contextualizing guideline recommendations involves:

- acknowledging the need for dialogue and formal consideration of local best available evidence and criteria for adopting, adapting or creating recommendations from an existing trustworthy source guideline to the national, local or other level;
- deciding whether the recommendations are suitable for that setting; and
- modifying or adding to the recommendations to optimize their implementation using structured and transparent processes.

These adjustments to context are necessary to support guideline implementation and can be relatively simple, for example, considerations about the target population when extrapolating recommendations from one country to another country with a similar context. Other times, more complex adaptation processes might be required, such as when cost and feasibility differ from the considerations made in the global guideline. For example, both nurses and physicians can perform cervical cancer screening, and the choice may depend on workforce availability in the country. Regardless of the degree of contextualization, recommendations must adhere to international standards for trustworthy guidelines.^5^^–^^9^

There are several frameworks for guideline adaptation, of which the GRADE Adolopment approach has been described as the most complete.^10^ The approach, documented in WHO’s *Strengthening countries’ capacities to adopt and adapt evidence-based guidelines: a handbook for guideline contextualization*,^3^ allows adoption and adaptation of WHO and other agencies’ evidence-based guidelines to other jurisdictions through a systematic and transparent contextualization process. Contextualization enables countries to use existing guideline recommendations and the evidence on which they are based, resulting in reduced costs and time required to develop their own guidelines while ensuring local ownership and relevance.^11^ The process can also identify the need for new recommendations when none exist.

This contextualization process requires country-level capacities and specific skillsets that not all countries possess and, therefore, they would benefit from guidance and support. Here, we suggest ways forward for the WHO European Region, building on a case example from Estonia.

## Estonia case study

For a health guideline to be officially recognized as a national guideline and to be implemented in Estonia, it must be developed using methods and processes set in the Estonian handbook for guideline development.^12^

In 2015, Estonia had one the highest incidence of human immunodeficiency virus (HIV) infection in the WHO European Region, with an incidence of 20.6 per 100 000 population.^13^ To address this problem, the Estonian ministry of social affairs initiated in 2016 the development of the national guidance on HIV testing and treatment in Estonia. One of the reasons for initiating this guideline was variation in practice for first-line HIV treatment and the lack of national strategy on the use of pre-exposure prophylaxis, despite existing global WHO recommendations.^14^^,^^15^

Before the start of the guideline development, the ministry of social affairs, in collaboration with professional society representatives and a coordinating centre for national guidelines (the Guideline Unit at the University of Tartu), narrowed the scope of the guidelines. Development began in January 2019, after the release of the updated WHO global guideline.^15^ Following discussions with relevant stakeholders, the ministry in collaboration with the Guideline Unit created a multistakeholder guideline development group. This group included an individual living with HIV; a family doctor; three infectious disease doctors; a representative responsible for procuring HIV medicines at the Estonian Health Insurance Fund; a representative overseeing programmatic aspects at the National Institute for Health Development; an officer responsible for HIV policies at the ministry of social affairs; and a WHO Country Office representative, who provided WHO support. The guideline development group was chaired by a content expert and supported by a methodologist from the Guideline Unit and an external consultant. The Estonian Guideline Advisory Board, the overseeing body, approved the guideline development group and the Estonian Health Insurance Fund funded the work. Group members had to stringently report conflicts of interest, and any interests reported were managed according to the handbook guiding the process.^12^^,^^16^ The guideline development group used the GRADE-Adolopment approach for the contextualization as suggested in the Estonian guideline handbook.^12^ All group members received up to 3 hours of training on methods, and the methodologist a 1-day training on the GRADE-Adolopment approach.

The guideline development group assessed the PICO (population or patient, intervention, comparison or control and outcome) questions in two source guidelines: *Consolidated guidelines on the use of antiretroviral drugs for treating and preventing HIV infection: recommendations for a public health approach,*^14^ and *Updated recommendations on first-line and second-line antiretroviral regimens and post-exposure prophylaxis and recommendations on early infant diagnosis of HIV*.^15^ The group also obtained evidence-to-decision and GRADE tables, systematic reviews, meta-analysis and search strategies associated with the PICO questions from the WHO responsible technical officer of the source guidelines. To assess resource use, the group used national health technology assessment reports from the Centre for Health Technology Assessment at the University of Tartu.^17^^,^^18^ The guideline development group also considered the most recent international guideline from the European AIDS Clinical Society.^19^ However, after appraisal of all source guidelines using the AGREE II tool,^20^ the guideline development group disregarded the guideline because the evidence informing the recommendations was neither clearly described nor were the developers willing to share it.^21^

The group prioritized only nine out of 35 PICO questions for the Estonian guideline (available from authors’ online repository).^22^ These questions focused on treatment aspects requiring consideration of local context, local evidence (for example, results of the local health technology assessment reports), and further discussion on treatment aspects between development group members. Of these prioritized questions, the question regarding the initiation of treatment from the WHO HIV consolidated guideline^14^ was changed to consider the entire drug class instead of only a single drug. This change was made because, in the Estonian context, recommendations limited to one drug are not applicable due to local price and procurement concerns. The group also added a question for treatment failure to answer a locally relevant priority question. Ultimately, evidence from the 10 PICO questions was used as one question used evidence from two PICO questions. After the guideline development group had finalized the scope and rating of the outcomes for their relative importance, a methodological support team of seven members, set up specifically for the guideline, updated evidence summaries and evidence-to-decision tables with findings from updated systematic review searches and local contextual evidence.

In total, the guideline development group held six 3-hour meetings. During these meetings the group rated outcomes, considered the source guidelines with updated evidence synthesis and supplemented them with the best available local data, and added their own expert evidence, where appropriate. The group members assessed relevant contextual factors, discussed evidence, implementation considerations and formulated recommendations. The guideline development group adopted several WHO recommendations without important changes (online repository).^22^ For example, the group strongly recommended pre-exposure prophylaxis and refined the target populations. The group also discussed the implementation practicalities, recording them in the implementation plan. Meanwhile, some recommendations were adapted due to the differences in feasibility, cost and resources. For instance, instead of recommending dolutegravir as first-line treatment, the national guideline^21^ recommends a combination of two nucleoside analogue reverse-transcriptase inhibitors and an integrase strand transfer inhibitor. In case of treatment failure, the national guideline recommends a viral resistance test before prescribing the second line regimen. While the WHO guideline did not recommend the test for routine use, considering it too costly and complex, the guideline development group regarded these issues as not applicable in Estonia, as the concerns over emerging viral resistance outweighed the cost concerns.

The GRADE-Adolopment approach allowed for flexibility in providing recommendations that addressed the most important questions of Estonian stakeholders, considering local priorities. This approach allowed the group to consider the context in which they lived and worked, integrating availability of local resources, acceptability, feasibility and equity into decision-making. Most importantly, the involvement of all relevant national stakeholders in the guideline development group facilitated the uptake of trustworthy recommendations, and resulted in subsequent policy and coverage decisions. This structured and participatory process also resulted in practical implementation decisions, actions and advice that reflected agreement between stakeholders on the roles, action or health-care arrangements. For instance, the process of including pre-exposure prophylaxis-related medicines in the list of reimbursed medicines was initiated before the guideline’s finalization, and the guideline recommendations were directly used in the central procurement of HIV medicines. Adapting the recommendation from a single drug in the WHO source guideline to a class-based listing in Estonia ensured competition among pharmaceutical providers, resulting in significant cost savings in purchasing antiretroviral medicines. The guideline was also complemented by an implementation plan and patient information material regarding pre-exposure prophylaxis. Similar processes and approaches were applied for National HIV testing guidelines,^23^ based on the WHO 2015 Consolidated Guidelines on HIV Testing Services.^24^

As an outcome of the contextualization process, the group developed the national guideline^24^ for pre- and post-exposure prophylaxis of HIV infection and treatment of people living with HIV in less than one year, compared with the usual timeline of up to two years. Using trustworthy materials from WHO, but still ensuring the usual participation and rigour of guideline development, significantly shortened the process and saved resources. Evidence for the uptake of the contextualized guideline comes from an observational study describing the use of pre-exposure prophylaxis in Estonia. The first prescription of pre-exposure prophylaxis was issued in May 2020, and a total of 23 prescriptions were issued in 2020. In 2022, the total number of prescriptions had increased to 156 (including 82 new initiations).^25^ These prescription data suggest that a national guideline may have contributed to initiating pre-exposure prophylaxis prescription in 2020. Although a programme evaluation indicates that health workers in Estonia prefer contextualized guidelines,^26^ we lack high-quality evidence demonstrating that contextualization consistently achieves a greater impact than providing global WHO guidelines, and conducting quantitative research to demonstrate impact in smaller countries is challenging. Thus, future evaluations of the Estonian guideline development programme could include qualitative work as recently conducted in Latin America.^27^ These evaluations could also be conducted in other jurisdictions to understand and optimize the impact of guideline contextualization.

## Lessons learnt

Our described country case has some key lessons for other jurisdictions. First, for those countries developing guidelines de novo, this contextualization approach saves time and resources while ensuring the benefits of country-specific information, such as on priorities and resources. Second, for those adapting guidelines, a structured and transparent framework for contextualization helps to ensure formal discussion for consensus building, context specificity and rigour of development. Third, given the small size of Estonia, with a population of 1.3 million people, this approach may be feasible in other smaller countries.

However, for a successful process, such as in Estonia, key requirements need to be met to overcome common guideline adaptation challenges.^28^ First, awareness and acceptance of high-standard methods and processes for guideline development are essential. The availability of necessary resources, capacities and processes is also key. In Estonia, these requirements were ensured through guideline institutionalization efforts since 2011.^12^^,^^16^ Second, methodological guidance and support are crucial, including investments in building methodological skills within the country, and the clarification and update of methods through a handbook for guidelines. Third, the availability of and access to high-quality source guidelines and background material are necessary.

Many countries in the WHO European Region, such as Germany, Italy, Spain and the United Kingdom of Great Britain and Northern Ireland, have strong capacity and exemplary guideline development programmes. In other countries, such as Czechia,^29^ progress has been made in modernizing guideline creation processes and strengthening the required capacities and institutions, but the situation across the region remains uneven. The WHO Regional Office for Europe’s rapid situation assessment in countries, conducted through individual and group exercises at a technical meeting in Copenhagen, Denmark in 2023 (attended by representatives of 21 countries of the 53 WHO European Member States) along with the experiences from country work in Estonia, Kyrgyzstan, Slovenia, Sweden, Tajikistan and Ukraine,^30^ revealed knowledge gaps regarding the complexities and nuances of developing, adopting and adapting trustworthy health guidelines. Persistent challenges exist to obtain and secure adequate resources, technical and methodological capacity and support structures for developing and implementing guidelines, as well as evaluating their impact. For example, to support guideline development in Tajikistan for hypertension and diabetes during pregnancy, the WHO Regional Office for Europe raised awareness and provided training and feedback to the stakeholders and guideline developers about the standards and methods of developing trustworthy guidelines, including the engagement of stakeholders, management of conflicts of interest, and systematic application of local evidence and methods. However, further support and progress are needed in the region to ensure that the processes are institutionalized and that there is sufficient local methodological capacity to support guideline efforts.

## Ways forward

To strengthen countries’ capacities to adopt and adapt evidence-based guidelines, we suggest four actions to support the Member States in the WHO European Region. First, standardize methods in guideline production to increase efficiency and improve quality. Countries benefit from guidance on structured and standardized methods for adaptation and adoption. The handbook *Strengthening countries’ capacities to adopt and adapt evidence-based guidelines: a handbook for guideline contextualization* aims to address this methodological gap.^3^ However, systematic adaptation approaches also depend on global efforts to improve the quality of potential source guidelines that adhere to the international standards for guideline development and reporting. Such adherence is essential for the successful adaptation of source guideline recommendations, as it requires rigour in development, clear reporting (such as evidence summaries in an easy-to-use and easy-to-translate format) and available relevant information (for example, complete evidence-to-decision tables and electronic files).

Second, acknowledge the critical role of continuous and shared learning. Capacity-strengthening emerged as an essential theme from the work the regional office conducted with countries. Guideline development and implementation requires specific skills and knowledge that are not always available. The guideline science is evolving with new methods, approaches, challenges, such as the use of artificial intelligence in guideline production, and tools which necessitate continued learning. Enhancing awareness, skills and knowledge of professionals involved in guideline development, as well as health workers, policy-makers and other stakeholders is vital for sustained progress in guideline development, contextualization and implementation. Facilitating and sharing experiences between countries provides a great opportunity to enable this progress. For example, the Estonian guideline programme served as a foundation for the model envisioned for Ukraine in 2021 (postponed due to the war and lack of resources). The regional capacity-building would benefit from a structured platform for collaboration and knowledge exchange among countries. Such a platform would not only facilitate learning and inspiration from others, but also provide an avenue for sharing and building new knowledge in guideline science. Additionally, such a platform could lead to capacity-strengthening, the co-creation of solutions and tools, and collaborative projects among Member States. This platform could be further enhanced by implementing a system for evaluation and feedback, along with methods for identifying and addressing potential barriers that may emerge during efforts to strengthen guideline development in countries. 

Third, guideline programmes and initiatives should be institutionalized. Most countries conduct some form of national guideline development, adaptation or implementation efforts. However, in many countries, the processes and methods are not fully established, integrated into the health system or aligned with international standards. Thus, support for institutionalization of guideline programmes at a country level is needed in WHO European Region. Given the diversity in health systems and resources across countries, tailored approaches are required. Countries, such as Kyrgyzstan, Slovenia, Tajikistan and early on Estonia and Ukraine, have contacted WHO for more individualized technical assistance in strengthening guideline programmes. To provide meaningful suggestions to these and other Member States on how to modernize an existing guideline programme, a comprehensive assessment of needs and mapping of the capacity in the region is required. Developing guidance for conducting a maturity situation analysis and establishing a governance framework that links with other health decision-making processes is crucial and planned for the WHO European Region.

Finally, provide support and identify resources. To better integrate contextualization into guideline development, joint efforts are needed to advocate for and negotiate with potential funders. Building a support system through training and certifying trainers and methodologists, establishing a joint roster of experts and leveraging expertise through WHO Collaborating Centres would further help sustain these efforts. Additionally, raising awareness of the contextualization approach among WHO guideline groups as well as WHO country office staff is essential. This sensitization will create closer collaboration between regional offices and WHO headquarters, ensuring alignment of different methodological approaches for adaptation and implementation. Such alignment will better account for country-level realities and needs, maximizing the potential of WHO guidelines to achieve local impact.

## Conclusion

Our work with countries, which varies greatly in available resources and capacity for guidelines, suggests that providing guidance and strengthening capacities in Member States for contextualizing guidelines developed for global or other settings is a unique, yet feasible way to enhance WHO’s normative leadership and guiding role. This capacity strengthening will increase the likelihood of implementing WHO guideline recommendations by improving their context sensitivity, relevance and appropriateness to the setting, while using the best available evidence and maintaining their credibility. This effort will also equip Member States with critical capacities and strong institutions to make context-specific evidence-based health decisions in an efficient manner. Strong recommendations and good practice statements may not require extensive contextualization, but having a formal platform for discussing and planning implementation adds clear value in developing recommendations that are relevant, acceptable and implementable. Having the institutionalized ability to develop contextually relevant guidance will become especially critical during future health threats such as pandemics and when addressing the health effects of climate change and conflict situations.

However, strengthening capacities, institutionalizing efforts and changing mindsets take time and requires substantial collaborative will, effort and resources. Recognizing these challenges there is also potential for increasing efficiencies and saving resources by facilitating sub-regional or small countries’ joint contextualization initiatives and improving methods for rapid contextualization. The outcomes of this area of work have the potential to increase trust and enable wise resource allocation, facilitating sensible and sustainable transformations in the health system. In the WHO European Region, we started to build on the insights gained from countries to foster collaboration and to continue adapting and refining methods to meet the evolving needs of these countries. Indeed, our work has already shown results, with the WHO Regional Office for Europe supporting Kyrgyzstan and Slovenia in reviewing and revising their guideline efforts, and Sweden in piloting contextualization of a WHO guideline.
